# Genetics of rheumatoid arthritis susceptibility, severity, and treatment response

**DOI:** 10.1007/s00281-017-0630-4

**Published:** 2017-05-29

**Authors:** Sebastien Viatte, Anne Barton

**Affiliations:** 1 Arthritis Research UK Centre for Genetics and Genomics, Centre for Musculoskeletal Research, Faculty of Biology, Medicine and Health, Manchester Academic Health Science Centre, The University of Manchester, Oxford Road, Manchester, M13 9PT UK; 2 NIHR Manchester Musculoskeletal Biomedical Research Unit, Manchester Academic Health Science Centre, Central Manchester University Hospitals NHS Foundation Trust, Grafton Street, Manchester, M13 9WL UK

## Abstract

A decade after the first genome-wide association study in rheumatoid arthritis (RA), a plethora of genetic association studies have been published on RA and its clinical or serological subtypes. We review the major milestones in the study of the genetic architecture of RA susceptibility, severity, and response to treatment. We set the scientific context necessary for non-geneticists to understand the potential clinical applications of human genetics and its significance for a stratified approach to the management of RA in the future.

## History

Although it is well recognised that ankylosing spondylitis, gout, and osteoarthritis have existed for several thousand years, the situation is less clear for rheumatoid arthritis (RA). Paleopathological evidence on skeletal remains dating back to the time of pre-Columbian Indians would suggest that RA is also an ancient disease, which might have been affecting the lives of people for at least 4000 years [1]. However, despite the expansion of archaeology and the accumulation of material to examine, the age and origin of RA are still a matter of debate and controversy, as the distinction of RA from other rheumatic diseases can rarely be made solely on the examination of bones [2]. Nonetheless, several indications from medical and non-medical literature and from the visual arts have suggested that RA has existed for many centuries, but it was not until 3 August 1800 that the unequivocal first diagnosis of RA was made by the French physician Landré-Beauvais of Paris [3]. He described in detail the clinical picture of the disease and originally called it “goutte asthénique primitive” (“primary asthenic gout”). His first nine patients were all women; an early suggestion of a genetic component to disease aetiology.

The nineteenth century saw the basic concepts of heredity and evolution being established with the publication of the theories of evolution by the British scientists, Charles Darwin and Alfred Wallace, in 1858 [4] followed by the discovery in 1865 of the laws of heredity by the Austro-German Augustinian Friar Gregor Mendel through his breeding experiments with peas [5]. These publications arose without any knowledge of the existence of DNA, which was only discovered later, in 1869, by a Swiss physician and biologist, Friedrich Miescher [6]. In this historical and scientific context, the observation that patients with RA aggregated occasionally in families supported the idea that the disease was at least partially heritable, even if it did not follow a Mendelian pattern of inheritance.

Research on the mechanisms of blood transfusion incompatibility and studies on skin transplant incompatibility followed by the discovery of the major histocompatibility complex (MHC) in the twentieth century further established the scientific and experimental context for ground-breaking discoveries in the genetics of RA. For example, the practice of mixing blood from two individuals before a transfusion to test their compatibility or the experimental practice of mixing only their lymphocytes to study their tissue compatibility or reactivity led to the development of so-called mixed lymphocyte cultures (MLC); in 1969, Gonzalo Astorga and Ralph Williams observed a reduced reactivity of lymphocytes from patients with RA when incubated together in MLC [7], indicating that the tissues from patients with RA were “more compatible” with each other than any pair of randomly selected healthy individuals—a further indication of a common genetic background between patients with RA. Further development of tissue typing using serological techniques in addition to MLC was used by Peter Stastny, who, between 1976 and 1978, described a strong association between MLC type Dw4 or serological type HLA-DRw4 and RA in white populations from the United States [8–10]. This association was further found to be present with a limited number of other specific types, namely, DR4 Dw14.1, DR4 Dw14.2, and DR4 Dw15.

In the 1990s, DNA-based techniques superseded serotyping and provided much greater precision. The MLC and serologic markers listed above were found to correspond to different alleles of the HLA-DRB1 gene, each coding for a different amino acid sequence. These alleles were subsequently renamed HLA-DRB1*04:01 (for DR4 Dw4) and HLA-DRB1*04:04, *04:08, and *04:05, for DR4 Dw14.1, DR4 Dw14.2, and DR4 Dw15, respectively.

## Definitions, techniques, and nomenclature

In animals and humans, the MHC region comprises a set of genes encoding proteins mainly involved in immune function. The human MHC is synonymous with the human leukocyte antigen (HLA) system, which lies on the short arm of chromosome 6 at position 6p21.3. The classical HLA region is highly variable (polymorphic) with, for example, over 1900 alleles for the HLA-DRB1 gene alone. A detailed description of the HLA region is available online through the European Bioinformatics Institute and the International Immunogenetics Project [11]. As the techniques used to type HLA genes have evolved over the years, the nomenclature for the different alleles at individual HLA genes has changed. Originally, immunological typing methods were used, including serotyping and cellular typing with MLC, later to be superseded by various DNA-based typing methods.

### Immunological typing

Outside of rheumatology, the earliest typing was performed using human sera reacting reproducibly to certain HLA types. Over time, more specific sera were identified, which could split one specificity into several. Then, MLC were developed to achieve even more precise typing; in order to achieve reproducibility, panels of reference cells were developed. As sera and cells required continual updating, WHO international histocompatibility workshops were held periodically. When a consensus was achieved on a new antigenic specificity, it would be assigned a new number with the designation “w” for “workshop.” As a specificity became widely accepted, the w would be dropped and a “permanent” identifier would replace it. For example, in one workshop, a newly identified specificity was given the name DRw4, and a few years later, it was revised to DR4. “DR” implied a serologically-based typing, and “D” a cellular typing. In this way, a single HLA specificity could be either DR4 Dw4 or DR4 Dw10.

### DNA-based typing

The use of sequence-specific oligonucleotide probes (SSOP) is the most commonly used method to determine HLA alleles. First, PCR amplification of a desired portion of DNA is performed, such as a part of the DRB1 gene. Then, the amplified section of DNA is probed with different non-radioactively labelled SSOPs, which discriminate between different alleles. This method is used in commercially available automated or semi-automated HLA typing systems in many laboratories. Another technique of DNA-based typing is often called next-generation DNA-based typing and refers to the sequencing of DNA over the region of interest. This technique has become much more affordable in recent years as the cost of sequencing has reduced. A third technique has recently been developed for research purposes [12]: typing with genotyping microarrays. First, the genotype of single nucleotide polymorphisms (SNPs) located within the HLA region is performed by using dense genotyping microarrays. As this will not allow determination of all SNPs across the HLA region, missing SNPs are then imputed in silico using reference panels of complete DNA sequences from individuals of the same ethnicity (e.g., from the 1000 Genome Project). The third step consists of imputing four-digit HLA alleles from SNP data.

### Current nomenclature

The WHO Nomenclature Committee for Factors of the HLA System revised the naming conventions for HLA alleles in 2010 [11, 13]. Each HLA allele is now named using a unique identifier, which always starts with HLA, followed by a hyphen, followed by the name of the gene (e.g., DRB1), an asterisk (*), and up to four sets of digits separated by colons (i.e., HLA-DRB1*XX:XX:XX:XX). All alleles have at least a four-digit code corresponding to the first two sets of digits. The first set of digits describes the allele group or type, which frequently corresponds to the serological type. The second set of digits defines a specific HLA protein within the allele group. A so-called four-digit HLA type completely and unequivocally determines the protein structure at the amino acid level. HLA identifiers which differ in the two first sets of digits will designate two molecules differing by at least one non-synonymous nucleotide substitution (i.e., one that changes the amino acid sequence). The third and fourth sets of digits are used to distinguish between non-coding nucleotide variations.

## Heritability of RA and subphenotypes

The clustering of RA cases within families has been a consistent observation across studies and can be measured either as λs, the sibling relative risk, or λr, the relative risk to first-degree relatives (parents, children, and siblings) [14]. Values for λs or λr have been reported to lie between 2 and 10, consistent with an increased prevalence of RA within the families of affected individuals as compared to the general population [15–20].

A complementary and more popular approach to measure the genetic contribution to RA susceptibility is to determine the proportion of the variance of the disease which is explained by genetic variations (i.e., the genetic contribution to the disease), also called disease heritability [14]. Several different methodologies have been proposed to estimate heritability of RA, but all have important shortcomings, which have resulted in large variations in estimates.

One method to calculate heritability is based on the comparison between disease discordance in monozygotic and dizygotic twins. The most cited publications using this method estimate a disease heritability of around 60% in the UK and Finnish populations [21, 22], whilst a Danish study published in 2002 found a heritability of 0% [23], revised to 12% in a larger sample in a follow-up study in 2012 [24]. Interestingly, one study calculated heritability separately for the two main subsets of RA (anti-citrullinated protein antibodies (ACPA) positive and negative disease) and found no difference in heritability between the two subsets (68% and 66%, respectively). A main limitation of twin studies is sample size, as the number of twin pairs available in these studies was modest.

A second method consists of estimating heritability from familial aggregation using large population registers; this method was applied in the Swedish total population and concluded that heritability of ACPA-positive RA is around 50%, but that heritability is only 20% for ACPA-negative RA [25]. This study is likely to represent the most reliable heritability estimates to date given the sample size tested.

A third and increasingly popular method to calculate heritability requires genome-wide genetic profiles from large numbers of unrelated individuals [26]; one technique, called genome-wide complex trait analysis (GCTA), has been successfully applied to several complex traits [27], but results from RA studies have produced highly varying estimates; for example, heritability was estimated to be 52% in one study [28] but 0% for ACPA-negative RA and 19% for ACPA-positive RA in another [29].

Heritability can also be calculated for RA severity or response to treatment in order to determine the quantitative role of genetic factors on disease course or response to specific interventions. By contrast to the calculation of heritability for disease susceptibility, where healthy individuals have to be incorporated in the study design as controls, heritability calculations are performed in RA cases only (for example, comparing globally the genetics of patients with erosive disease with that of patients without erosions). Using such an approach, one study estimated the heritability of erosive disease in the Icelandic population to be 50% [30]. Three studies have attempted to calculate the heritability of response to anti-TNF treatment in RA in different populations: [31] found a heritability of 45% for the post-treatment reduction in the 28 joint Disease Activity Score (DAS28) and 60% for the change in Swollen Joint Count (SJC); Umićević et al. [32] reported a heritability of 71% for the change in DAS28 and 87% for change in SJC; however, Sieberts SK et al. [33] found only an 18% heritability for non-response, but no genetic contribution to the prediction of response. These results should be interpreted with caution, not only due to the limitations inherent to the calculation of heritability but also because there is no clear definition of “severe” disease or “response” to treatment.

In summary, large variations in the calculation of heritability are problematic as researchers cannot know how many more genetic factors for RA susceptibility, severity, or treatment response are yet to be identified, and therefore how much effort and money to further invest in susceptibility, severity, or treatment response gene identification.

## Susceptibility

The genetics of RA susceptibility has already been covered in detail in many reviews [34–40] and the most comprehensive meta-analysis of all available GWAS datasets has now identified over 100 loci associated with RA susceptibility [41]; rather than presenting an exhaustive listing of genetic loci associated with RA susceptibility, we will summarise the key findings necessary to understand the translational potential of genetics and the future directions of research.

### HLA

In the late 1970s and early 1980s, several studies identified associations between RA susceptibility and different alleles of the HLA-DRB1 gene. Gregersen and colleagues [42] formulated a unifying hypothesis in 1987 based on the observation that all associated alleles had a 5-amino acid sequence within the third hypervariable region of the DRB1 gene at amino acid positions 70 to 74 which were either ^70^QRRAA^74^, ^70^QKRAA^74^, or ^70^RRRAA^74^; this sequence was referred to as the “shared epitope”. The amino acids are located within the peptide-binding groove of the HLA-DRβ1 protein, an observation which strongly implicated antigen-presentation in disease aetiology.

The advent of large scale genotyping using microarrays and the collaboration of researchers in large international consortia meant that by 2011, data was available from over 5000 seropositive (i.e., ACPA or rheumatoid factor positive) RA cases and almost 15,000 unaffected controls, allowing an unprecedented resolution and the refining of the shared epitope hypothesis by Raychaudhuri and colleagues [43]. The strongest genetic association with RA susceptibility was found with the amino acid valine at position 11 (or a histidine at position 13) of the HLA-DRB1 gene. Amino acids at positions 71 and 74, located within the original shared epitope motif, represented secondary and tertiary independent effects, respectively. Previously reported association with other amino acid positions (for example with positions 67 [44], 70, 72, 73) were not found to be independent of positions 11/13, 71, or 74. Although the sample size of this study was large, power was still insufficient to distinguish between the effects of positions 11 and 13, since the carriage of a specific amino acid at position 11 (e.g., a valine) almost unequivocally determined the amino acid carried at position 13 (e.g., a histidine) and vice versa, due to the high linkage disequilibrium between them. Studies in larger sample sizes or in different ethnicities are required to disentangle the effects of these two positions.

Interestingly, the various amino acids that can be carried at each position can be ordered hierarchically according to the size of their effect on susceptibility. For example, although the carriage of a leucine at position 11 increases the risk of developing RA, the carriage of a valine increases that risk much more, whilst the carriage of a serine reduces the risk [43]. As one healthy person can carry an amino acid increasing the risk at one specific position (e.g., valine at 11), but protective amino acids at other positions (e.g., glutamic acid at 71 and leucine at 74), the overall genetic risk conferred by HLA-DRB1 for an individual can only be determined after taking the combination of the three HLA-DRB1 positions on a single chromosome into consideration. Such a combination is called a haplotype. Based on HLA-DRB1 positions 11, 71, and 74, only 16 different haplotypes exist within the Caucasian population (Table 1) and only eight of these are frequent (occurring in more than 5% of the population).

**Table 1:** Sixteen HLA-DRB1 haplotype classification based on amino acids at positions 11, 71, and 74

Position 11	Position 71	Position 74	Haplotype name	Classical HLA-DRB1 alleles	Haplotype frequency (%)	OR for RA susceptibility	OR for RA severity
**Valine**	**Lysine**	**Alanine**	**VKA-haplotype**	*04:01	**11**	**4.4**	**1.8**
**Valine**	**Arginine**	**Alanine**	**VRA-haplotype**	*04:08, *04:05, *04:04, *10:01	**6**	**4.2**	**1.8**
**Leucine**	**Arginine**	**Alanine**	**LRA-haplotype**	*01:02, *01:01	**11**	**2.2**	**1.5**
Proline	Arginine	Alanine	PRA-haplotype	*16:01	1	2.0	–
Valine	Arginine	Glutamic acid	VRE-haplotype	*04:03, *04:07	1	1.6	–
Aspartic acid	Arginine	Glutamic acid	DRE-haplotype	*09:01	1	1.6	–
Valine	Glutamic acid	Alanine	VEA-haplotype	*04:02	1	1.4	–
Serine	Lysine	Alanine	SKA-haplotype	*13:03	1	1.0	–
**Proline**	**Alanine**	**Alanine**	**PAA-haplotype**	*15:01, *15:02	**14**	**1.0**	**1.0**
**Glycine**	**Arginine**	**Glycine**	**GRQ-haplotype**	*07:01	**13**	**0.9**	**1.0**
**Serine**	**Arginine**	**Alanine**	**SRA-haplotype**	*11:01, *11:04, *12:01	**10**	**0.9**	**1.1**
Serine	Arginine	Glutamic acid	SRE-haplotype	*14:01	3	0.8	–
Leucine	Glutamic acid	Alanine	LEA-haplotype	*01:03	0.4	0.7	–
Serine	Arginine	Leucine	SRL-haplotype	*08:01, *08:04	3	0.7	–
**Serine**	**Lysine**	**Arginine**	**SKR-haplotype**	*03:01	**13**	**0.6**	**0.9**
**Serine**	**Glutamic acid**	**Alanine**	**SEA-haplotype**	*11:02, *11:03, *13:01, *13:02	**11**	**0.6**	**0.7**

Hierarchical classification of HLA-DRB1 haplotypes based on their effect size for susceptibility to RA [43] and for severity (presence of erosive disease [45]). Haplotype names are derived from one-letter amino acid codes. The classical shared epitope alleles correspond to the VKA-, VRA-, and LRA-haplotypes. The column “*Classical HLA-DRB1 alleles*” shows only some examples of 4-digit HLA types corresponding to the new classification. High-frequency haplotypes (≥5% general Caucasian population) are indicated in *bold*. Effect sizes are only given for these haplotypes for severity, as the study by Viatte et al. [45] was underpowered to evaluate accurately effect sizes of low frequency haplotypes

Similarly, a fourth smaller but statistically significant independent effect has been detected within the HLA region in patients with seropositive RA at HLA-B position 9, a fifth effect at HLA-DPB1 position 9 [43], and a sixth effect at HLA-A position 77 [46]. All six amino acid positions are located within the peptide-binding grooves of four different HLA molecules. This observation implicates antigenic peptide presentation to T cells as key to disease causation [47]. The presence of class I and II HLA allelic associations links both CD8^+^ and CD4^+^ T cells to the aetiology and pathogenesis of RA.

Studies in seronegative RA have identified associations at HLA-DRB1 amino acid position 11 (but not 71 or 74) and HLA-B position 9 within the peptide-binding grooves, corresponding to HLA-DRB1*03 and HLA-B*08 [46, 48]. Although HLA-DRB1 position 11 is shared between ACPA^+^ and ACPA^−^ RA, the effects of individual amino acid residues are distinct; for example, a serine is protective for the development of ACPA^+^ RA, but increases the risk of developing ACPA^−^ RA, whilst a glycine is protective for both subtypes. These observations confirm that ACPA^+^ and ACPA^−^ RA are two genetically distinct entities and suggest that separate peptide autoantigens may be implicated in their pathogenesis.

### Non-HLA

Prior to 2007, candidate gene association studies had identified few genetic susceptibility loci for ACPA^+^ RA. However, those that were identified conferred large effect sizes. For example, association with a missense polymorphism of the PTPN22 gene with RA was widely replicated in all populations in which the variant was present at a reasonable frequency [49]; interestingly, no association was found in East Asian populations where the variant is rare.

**Fig. 1:**
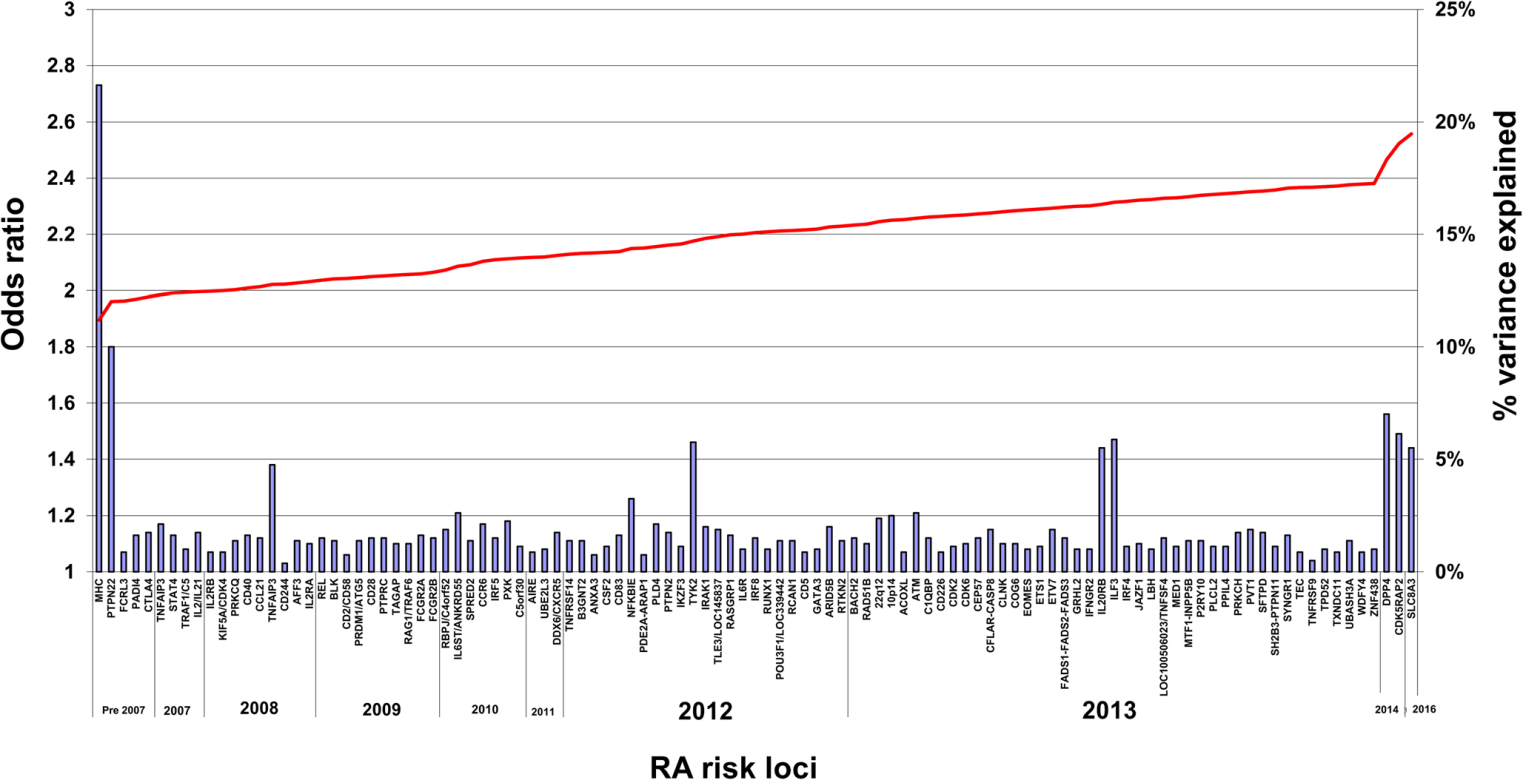
Cumulative proportion of the observed variance in rheumatoid arthritis susceptibility explained thus far by genetic susceptibility loci identified to date across Asian and Caucasian cohorts. Odds ratios (*left axis*) for RA genetic susceptibility loci are presented in the approximate chronological order of discovery (new associations from the study by Okada [41] are shown in 2013). The proportion of the variance explained (*right axis*) is indicated by the *black line*. A 0.5% disease prevalence was assumed for calculation. In the beginning of 2017, approximately 19.5% of phenotypic variance had been accounted for genetically. On average, every SNP outside the HLA explains 0.08% of the total phenotypic variance. For simplification, every locus is represented once, even if multiple independent effects were identified (except for TNFAIP3 and TRAF1/CDK5RAP2, where two independent effects are reported). The OR for the MHC represents the largest OR for a SNP across the MHC, but the % explained variance has been calculated for multiple independent effects across the MHC. Abbreviation: *RA* rheumatoid arthritis, *OR* odds ratio

The advent of genome-wide association studies (GWAS) in 2007 allowed the discovery of a large number of non-HLA markers for ACPA^+^ RA; large international consortia and meta-analysis permitted a massive increase in sample size and power in Caucasian and Asian populations [50, 51]. This approach culminated in 2014 with the publication of a transethnic mega-meta-analysis of all available GWAS datasets worldwide, comprising data from over 100,000 subjects of European and Asian ancestries (29,880 RA cases and 73,758 controls) for around 10 million SNPs [41]. Since then, further RA susceptibility risk loci have been identified (e.g., BACH2 [52], 22q12 [53], CDK5RAP2, and DPP4 [54], SLC8A3 [55]), bringing the total number of associations outside the HLA to 106 (Fig. 1). On average, each individual non-HLA locus explains 0.08% of the variance of the disease, and cumulatively, all risk loci, including those located within the HLA, only explain 19.5% of the disease variance, or 39% of disease heritability (assuming 50% heritability). Apart from HLA associations and PTPN22, most risk alleles harbour small effect sizes, with odds ratios for risk alleles between 1.01 and 1.20.

The 106 ACPA^+^ RA susceptibility loci discussed are all considered to be confirmed associations because the statistical evidence for association (*p* value) was below the threshold for claims of genome-wide significance (<5 × 10^−8^). Fewer robust statistical associations have been reported for ACPA^−^ RA, most likely because sample sizes are in general smaller for this more heterogeneous disease entity. Most genetic associations have been reported in single studies and have not been replicated independently. Four GWAS for ACPA^−^ RA were unable to identify any non-HLA association below genome-wide significance [48, 54, 56, 57]. Indeed, ANKRD55 is the only locus to be associated with ACPA^−^ RA at genome-wide significance levels [58, 59], and is also associated with other autoimmune diseases, including ACPA^+^ RA, juvenile idiopathic arthritis [60], and multiple sclerosis [61]. These studies have revealed that, whilst ACPA^−^ RA and ACPA^+^ RA are two genetically distinct subsets of RA, each with its specific set of susceptibility polymorphisms, they also share several genetic associations. For example, AFF3, CCR6, CCL21, IL2RA, and CD28 are associated with ACPA^+^ RA susceptibility but not with ACPA^−^ RA, whilst markers at TNFAIP3, C5orf30, STAT4, ANKRD55, BLK, and PTPN22 are associated with both serotypes [62]. By contrast, CLYBL [48], SMIM21 [48], SPP1 [63], CLEC16A [64], IRF5 [65], DCIR [66, 67], LEMD2, CSMD1, FCRL3 [57], IL-33 [68], PRL [59], and NFIA [59] have been reported to be associated with ACPA^−^ RA (although not at genome-wide significance thresholds), and many of these markers are not associated with ACPA^+^ RA.

### Variations across ethnicities

Ethnogenetic heterogeneity in RA exits [69] with genetic associations specific to one population. Cardinal examples include the association of HLA-DRB1^*^09:01 with RA susceptibility in Asian populations and PTPN22 in Caucasian populations.

Although the frequencies of four-digit HLA-DRB1 alleles are highly variable across populations, recent large scale studies have demonstrated that the same amino acid residues and positions confer risk to ACPA^+^ RA in Asian and European populations [70]. Though positions 11 and 13 are tightly linked in European populations, position 13 is the strongest association with RA susceptibility in Asian populations [70], and the observed difference in association from European populations was explained mainly by DRB1^∗^09:01. Studies in African Americans also identified position 13 as the main association with RA susceptibility [71].

The main reason for differential effects across populations of different ancestries (within the HLA or outside the HLA, e.g., PTPN22) is a variation of the allele frequency: the lack of associations in a specific population can be explained by the monomorphism of the locus in that population or by a very low allele frequency, prohibiting sufficient power to detect the effect. In general, the overwhelming majority of RA susceptibility loci are shared across populations [37, 38, 41].

### Insights into pathogenesis

The functional characterisation of the mechanisms of actions of RA susceptibility SNPs in disease causation has proved to be a challenging task, as only a minority of SNPs affect the protein coding sequence (e.g., HLA or PTPN22 SNPs). The vast majority of genetic susceptibility variants are located outside coding sequences or in gene deserts. It has been suggested that susceptibility variants exert their effect by disrupting the function of unknown DNA elements (for example, as yet unidentified enhancers). In addition, for a number of susceptibility alleles, the reported risk locus is likely representing a highly correlated proxy for the as yet unidentified causal allele. As a result of these caveats, the gene name assigned to a risk locus is frequently the closest or most compelling biologic candidate gene, although there might not be any direct evidence that its function is disrupted by the risk allele. Despite these limitations, GWAS have nonetheless identified pathways likely to be involved in RA pathogenesis, such as the CD40 signalling pathway (with RA susceptibility SNPs mapping close to the CD40, TRAF1, TRAF6, TNFAIP3, NF-κB (c-Rel) genes) or the T cell receptor (TCR) signalling pathway (PTPN22, RasGRP, PKC-θ, TNFAIP3, TRAF6, etc.) [37]. With HLA-DRB1 expressed on antigen-presenting cells (APC), the interaction between APCs and CD4^+^ T cells is likely to play a central role in the pathogenesis of the disease.

Several experimental strategies may be used to systematically identify the target genes and target cells of RA susceptibility SNPs: (A) the identification of genes, the expression level of which is correlated with the presence of a specific SNP. Such SNPs are called expression quantitative trait loci (eQTLs). eQTL studies have allowed the identification of the target genes of several RA susceptibility loci [72]; (B) the study of chromatin marks (epigenetics) overlapping susceptibility variants and the integration of gene expression patterns in different cell types has allowed assignment of certain SNPs to certain cell types [73, 74]; and (C) molecular techniques (for example Capture Hi-C) have been used to characterise chromatin conformation and identified long-range interactions between genetic variants associated with RA and their functional targets in B and T cell lines [75].

So far, these studies [72–79] have concluded that (1) many susceptibility variants may not interact with the nearest gene, but with genes situated several megabases away; (2) the effects of genetic variants are context-specific, i.e., will vary according to the cell type and stimulatory conditions present; and (3) regions associated with different autoimmune diseases interact with the same promoter, which suggests common autoimmune gene targets.

## Severity

The identification of genetic markers of RA outcome is a much more complicated task than the identification of susceptibility markers, as several methodological challenges have to be overcome. First, the definition of disease severity is not standardised; second, disease outcome varies over time; third, the sample sizes in prospective cohorts of patients with good quality longitudinal data on disease outcome are modest and, finally, statistical modelling is complex, as several outcome variables are continuous, non-normally distributed and affected by time-varying confounders, including treatment.

Despite these challenges, multiple studies have identified HLA-DRB1 alleles as markers of radiological damage in RA [80]; for example, a well-powered study has recently shown that the risk hierarchy defined by the 16 HLA-DRB1 susceptibility haplotypes (i.e., defined by positions 11/13, 71, and 74, *See* Table 1) was correlated between disease susceptibility, erosive damage, and mortality: thus, the major genetic markers of disease susceptibility in the HLA-DRB1 gene are also markers of severity [45]. Valine at position 11 is the strongest genetic predictor for the development of erosions, radiographic damage, mortality, and poor outcome in general, including non-radiographic measures of disease activity/outcome [45, 81]. A serine at the same position is protective against radiographic damage and poor outcome [45, 81]. One interesting observation was that effect sizes for disease outcome were systematically smaller than those observed for susceptibility (Table 1). Classification of patients with RA into different prognostic categories could be performed using HLA-DRB1 susceptibility markers, but the proportion of the variance of radiographic damage explained by HLA markers remains too low to be clinically useful.

**Fig. 2:**
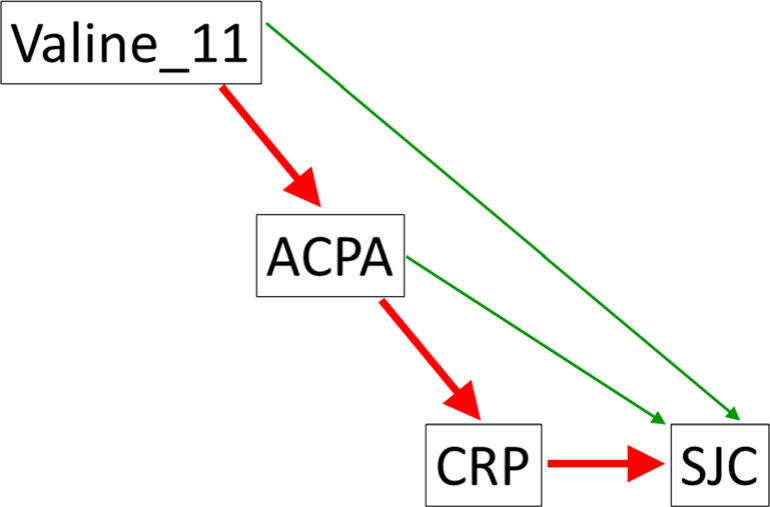
Major (*red*) and minor (*green*) pathways explaining the statistical association between genetic markers of clinical outcome variables in RA (acyclic graphs from mediation analysis [81]) (Color figure online)

Although most of the effect of HLA-DRB1 on disease severity is mediated by ACPA, there is some evidence emerging that HLA-DRB1 amino acids may regulate the level of laboratory inflammation (as measured by CRP) and clinical inflammation (disease activity score at 28 joints (DAS28) or Swollen Joint Count) through different biological pathways, some of which are likely to be independent of ACPA [45, 81] (Fig. 2).

Few GWAS have been performed for RA severity [82, 83], and most studies reporting genetic associations with radiographic outcome are candidate gene association studies (Table 2). Very few studies have reported associations below genome-wide significance for loci located outside the HLA region [83], and the replication rate of these associations in independent datasets has been very low [83]. Apart from HLA loci and SNPs located near TRAF1, which have been consistently associated with radiographic damage and replicated in several independent cohorts by independent research groups across different populations, no other genetic locus can be regarded as a confirmed association with radiographic outcome in RA, as replications by independent groups of researchers in large cohorts are lacking. Interestingly, a few RA severity SNPs have been followed up in functional studies to increase confidence that they are true positive associations and to understand the pathogenesis of severity: a SNP (rs12212067: T>G) in the FOXO3A gene region, which is not associated with RA susceptibility, has been reported to be associated with disease course in several TNF-mediated conditions, including RA [94]. The mechanism of action involves a reduction in the production of pro-inflammatory cytokines, including TNFα, by monocytes. The differential response of monocytes in RA patients dependent on the carriage of the minor allele at FOXO3A is likely to be seen only in an inflammatory context, therefore explaining the lack of association with disease susceptibility.

**Table 2:** Genetic associations with RA outcome

Gene name	Reference	Comment
*HLA-DRB1*	[45, 84–87]	
*TRAF1*	[88–92]	Not associated in [93]
*FOXO3*	[94, 95]	Not associated in [96]
*C5orf30*	[97]	Mechanism of action of the SNP studied in [98]
*IL2RA*	[99, 100]	
*IL2RB*	[100]	
*SPAG16*	[82]	GWAS
*CD40*	[101]	
*TNFAIP3*	[102, 103]	Not associated in [93]
*TNF-α*	[104]	
*IL-4 receptor*	[105, 106]	
*IL-4*	[107]	Not associated in [105]
*DKK-1*	[108]	
*MMP-9*	[109]	
*ZFP36L1 / C14orf181*	[109]	
*Granzyme B*	[110]	
*CCR5*	[111]	
*FCRL3*	[112]	
*IL15*	[113]	Not associated in Japanese [114]
*PADI4*	[84]	
*LILRA3*	[115]	
*SPP-1*	[116]	ACPA-negative RA
*Osteoprotegerin*	[117]	
*HUNK / SCAF4*	[83]	GWAS in ACPA-negative RA
*PTGER4*	[118]	
*CRP*	[119]	

We list here some genetic associations with radiographic outcome in RA reported in the last 5 years (since 2012). Associations reported previously have been reviewed by Marinou et al. [120] and Viatte & Barton [80]. Gene names have been assigned to genetic polymorphisms based on the most plausible biological candidate or nearest gene. Genetic associations with other measures of disease severity are not presented here; non-radiographic measures of disease outcome are usually more noisy (less stable or reproducible) than radiographic measures, which make them less suitable for genetic studies, where available cohorts have a modest sample size

The identification of the association of rs26232, a SNP located in the first intron of the chromosome 5 open reading frame 30 (C5orf30), with both RA susceptibility and severity, has prompted the functional characterisation of C5orf30, a gene with previously unknown functions [98]. C5orf30 was found to be expressed at high levels in the synovium of patients with RA compared with control synovial tissue. C5orf30 decreases the migration of synovial fibroblasts, so that loss of function increases joint inflammation and tissue damage [98].

The two examples of FOXO3A and C5orf30 illustrate the potential of genetic studies in the identification of important pathogenetic mechanisms of RA susceptibility and severity. Although our current understanding of the genetics of RA severity is much more limited than our understanding of the genetics of RA susceptibility, the emerging picture seems to be that there is only a partial overlap between genetic markers of susceptibility and severity. Moreover, it is also likely that genetic markers of severity are different between ACPA^+^ and ACPA^−^ RA [83, 116].

## Treatment response

Identifying biomarkers to target the right treatments to the right patients would bring immediate patient benefit in RA because a number of treatment options exist and some patients will do well with each option whilst, in others, disease activity will remain uncontrolled leading to impaired quality of life for the patient and increasing the risk of long-term disability or will result in adverse effects in some patients [121, 122]. In addition, some of the treatment options are expensive thereby impacting on healthcare resources; for example, biologic drugs targeting inflammatory pathways cost between £5 and 10K per year per patient. If a stable biomarker could be used to select the best treatment option for individuals or groups of patients with RA, it would have the potential to improve health and costs of healthcare, simultaneously. Hence, RA is an ideal condition in which to apply such precision medicine approaches. Genetic biomarkers are stable and are easily assayed. In other disease areas, genetic biomarkers are being used to inform treatment selection decisions; for example, EGFR gene mutation screening in non-small cell lung cancer is undertaken to determine suitability for oral tyrosine kinase inhibitors (reviewed in Shea et al. [123]). However, no genetic variants have yet been robustly and consistently associated with response to therapies used in RA. Methotrexate is the most common first-line disease modifying drug choice, but results from candidate gene association studies have been conflicting and few GWAS have been undertaken to date [124, 125]. Similarly, biologic drugs targeting the TNF pathway (THF inhibitor (TNFi) drugs) are the most common first choice biologic treatment, but results of genetic association studies have often been conflicting. For example, an association of PDE3A-SLCO1C1 at genome-wide significance levels with TNFi response has been reported but not replicated [126, 127], whilst association of the PTPRC gene polymorphism has been associated with TNFi response in some [128–130] but not all studies [131, 132].

The lack of success in identifying treatment response biomarkers is disappointing, but not unexpected, given that the studies face many of the same challenges as for disease severity studies: First, the outcome measure is a composite of both objective and subjective measures making reliable, consistent, and standardised measurement difficult. Furthermore, many of the subcomponents of the outcome scores are based on clinical assessments, which may not be truly reflective of response in terms of synovial inflammation. For example, the DAS28 score comprises a clinical assessment of 28 joints for swelling and tenderness, a serological marker of inflammation (ESR or CRP), and a score of global well-being provided by the patient; changes in the DAS28 score before and after treatment are used to assess treatment response. Third, disease outcome varies over time; fourth, the power of such studies is limited by sample size. To illustrate the latter, the most comprehensive study of RA susceptibility loci involved analysis of samples from over 100,000 individuals whereas the largest analysis of TNFi response was based on ~2700 patients [41, 133]. Finally, other factors, such as whether the patients actually take the drug prescribed (adherence) or whether antibodies to the drug develop have very important influences on response, but are not yet accounted for in studies [134–136].

Given that genetic variants will act on specific biologic pathways, it is likely that genetic studies may be better correlated with changes in synovial inflammation, as that is the target of such treatments; however, synovial inflammation is poorly correlated with DAS28. Therefore, re-weighting of current measures or, better, new biological outcome measures are required that better reflect the synovial inflammatory response in order to better classify responders and identify factors that predict response pre-treatment. For example, giving higher weightings to the components of the DAS28 score that correlate better with synovitis (the swollen joint count and serological inflammatory markers) has been proposed [137], whilst others have used DCE-MRI scans to accurately quantify synovitis to determine treatment response [138].

## Clinical utility and perspectives

Clinical prediction models incorporating genetic susceptibility loci to identify healthy individuals at high risk of disease have shown a very modest prediction performance and are insufficiently accurate for general population screening [139]. Also, genetic markers are not recommended for diagnosis. The clinical utility of a genetic stratification system for precision medicine based on HLA haplotypes correlated with disease course or outcome [45] remains to be evaluated, but its performance is likely to be equivalent or inferior to ACPA status, thus insufficient to guide clinical decisions, as the association of HLA with severe disease acts mainly through the presence of ACPA. Currently, the testing of patients for HLA-DRB1 is therefore only performed as a research tool. However, further methodological developments and the identification of an increasing number of susceptibility/severity/treatment response SNPs are ongoing. Together with the identification of other types of biomarkers (epigenetic, immunological, cellular, serological …), genetic markers might allow in the future the definition of combined genetic, demographic, laboratory, and clinical risk scores to accurately classify patients at diagnosis into different prognostic or treatment response categories for precision medicine.

## Conclusions

GWAS have been extremely successful in the identification of a large number of genetic susceptibility polymorphisms associated with RA. Although the effect sizes of SNPs outside the HLA are modest, genetics has shed new light on pathogenetic mechanisms of disease susceptibility and has been hypothesis generating. Functional genomics approaches are now taking over from genetic association studies to identify the mechanisms of actions of susceptibility polymorphisms. The identification of genetic markers of disease outcome and response to treatment is still at its infancy, but bears the potential to contribute to the development of a precision medicine approach in the management of RA in the next 10 years.
